# Multivalent Ion-Conducting Metal- and Covalent- Organic Frameworks

**DOI:** 10.1021/acsaem.5c01925

**Published:** 2025-09-11

**Authors:** Zhilin Du, Wonmi Lee, Dawei Feng

**Affiliations:** † Department of Chemistry, 5228 University of Wisconsin − Madison, Madison, Wisconsin 53706, United States; ‡ Department of Materials Science and Engineering, University of Wisconsin–Madison, Madison, Wisconsin 53706, United States; § Department of Chemical Engineering, 567045 Kongju National University, 1223-24 Cheonan-Daero, Seobuk-gu, Cheonan, Chungnam 330-717, Republic of Korea

**Keywords:** multivalent-ion conductors, metal organic frameworks, covalent organic frameworks, solid electrolytes, cost-effectiveness

## Abstract

Metal–organic frameworks (MOFs) and covalent organic frameworks (COFs) offer uniquely tunable nanoporous architectures, and ionic groups can provide the additional hopping sites, rendering them promising ion conductors for multivalent-ion batteries (e.g., Zn^2+^, Mg^2+^, Ca^2+^, Al^3+^). This review first examines the unique structures and ion transport mechanisms, highlighting how framework flexibility and functionalization lower activation energies for bulky multivalent cations. We then outline structural design principles, including incorporation of ionic groups to maximize ionic conductivity. Key synthetic methods such as mechanical grinding, ball milling, reflux, hydrothermal/solvothermal, and interfacial synthesis are compared in terms of crystallinity, scalability, and environmental impact. Potential applications of MOF/COF as solid electrolytes, membranes, and interfacial coatings for multivalent batteries to improve cycle life. The future research directions are also proposed to enable MOF/COF materials as practical conductors in next-generation multivalent-ion energy storage systems.

## Graphical abstract

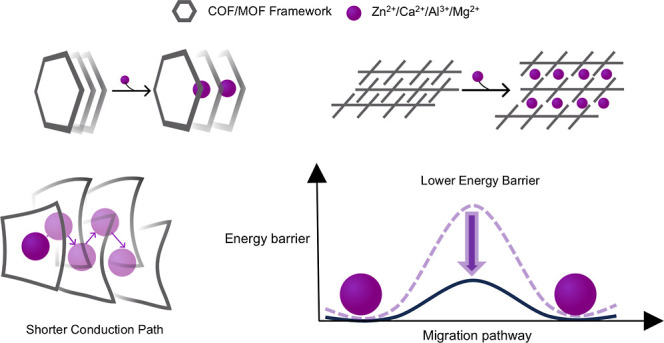

## 1 Introduction

Ion conductors have attracted considerable interest owing to their diverse applications in batteries, fuel cells, electrochemical sensors, and other energy-conversion devices.^1–10^ In battery systems in particular, ion conductors serve as solid electrolytes in solid-state batteries and as selective membranes in flow batteries.^1–4^ The rapid development of solid-state batteries in driven largely by increasing demands for safe, high-performance energy storage in electric vehicles and stationary applications. The design and synthesis of ion conductors with high ionic conductivity, wide electrochemical stability windows, and excellent interfacial compatibility with electrode materials are pivotal to the commercialization of solid-state batteries.^1,2^

Research on Li^+^-conductors is extensive, as small ionic radius of Li^+^ ion among alkali metals affords a low migration energy barrier.^11–17^ Inorganic oxides and sulfides, for instance, demonstrate ionic conductivities of 10^–3^–10^–2^ S cm^–1^, excellent electrochemical stability, and high mechanical integrity; however, they exhibit substantial grain-boundary resistance, inherent brittleness, and require high-temperature sintering.^11–14^ Polymer electrolytes—such as PEO- and PVDF-based systems—provide flexibility and low-temperature processability, and their ionic conductivities can be enhanced via additive incorporation.^15–17^ Nonetheless, their limited conduction pathways and relatively low mechanical strength impose performance constraints.

Although several inorganic and polymer materials show promise as ion conductors for monovalent-ion solid-state batteries, research on multivalent-ion conducting materials remains limited. Multivalent batteries are gaining attention as next-generation secondary batteries due to their numerous advantages.^18,19^ First, they offer high theoretical volumetric energy density due to multielectron redox reactions and the higher true density of multivalent metals compared to monovalent metals. Second, multivalent metals such as Mg, Al, Ca, and Zn are earth-abundant and significantly cheaper than Li or Na. Third, these metals are thermally stable for electrolytes including aqueous electrolytes, greatly reducing the risk of thermal runaway or electrolyte combustion. Additional benefits include extended cycle life and well-established recycling processes, further highlighting their potential as sustainable, high-energy-density secondary batteries.

However, developing highly conductive materials for multivalent ions is more challenging than for Li^+^, Na^+^, or K^+^ due to the larger ionic radii and stronger Coulombic interactions.^20^ Conventional inorganic or polymer electrolytes often suffer from insufficient voids for these bulkier ions, resulting in reduced conductivities.^21–25^ To address this limitation, research has turned to organic framework materials—such as metal–organic frameworks (MOFs) and covalent organic frameworks (COFs)—which offer well-ordered channels and tunable functionalities that lower activation barriers for larger cations.^26–36^ Moreover, the pore sizes of MOF/COF structures can be precisely adjusted to enhance ion selectivity, and their architectures can be diversified by selecting various organic linkers, metal nodes or functionalized monomers.^37,38^ Consequently, their tunable nanopore channels and functionalizable pore environments can lower ion migration barriers and enhance conductivity. As shown in Table 1, MOF and COF electrolytes can achieve higher multivalent-ion conductivities and lower activation energies than inorganic or polymer materials.^39–47^

**Table 1:** Ion Conductivities of MOF/COF Than Inorganic or Polymer Materials for Multivalent Ions

type	chemical formula	σ (mS cm^–1^)	*E*_a_ (eV)	transference number	ref
MOF/COF	TPB-PEO-9-COF-Mg	0.18	0.32	0.31 (Mg)	^34^
	Mg_2_DOBDC	0.25	0.11–0.19	NA	^35^
	MgI_2_@TB-MOF	0.328	0.21	0.76 (Mg)	^36^
	ZnI_2_@TB-MOF	0.61	0.20	0.84 (Zn)	
	CaI_2_@TB-MOF	0.13	0.19	0.77 (Ca)	
	MOF-MgCl_2_	0.012	0.32	NA	^46^
	MOF-MgBr_2_	0.13	0.24	NA	
	MIL-101⊃{Mg(TFSI)_2_}_1.6_	0.8	0.18	0.41 (Mg)	^47^
Inorganic	MgZr_4_(PO_4_)_6_	0.029	0.83	NA	^37^
	MgSc_2_Se_4_	0.1	0.37	NA	^38^
Polymer	Mg(CF_3_SO_3_)_2_	0.17	0.21	NA	^39^
	Zn(CF_3_SO_3_)_2_	0.21	0.16	NA	
	Zn(TFSI)_2_-acetamide	0.011	0.56	0.46 (Zn)	^40^

In this review, we compare the ion-conducting mechanisms of MOF/COF frameworks with those of inorganic ion conductors, highlighting their unique structural features. Previous review articles have primarily focused on the use of MOF and COF materials in monovalent-ion batteries.^48,49^ In contrast, this review emphasizes their application in multivalent-ion batteries, with a particular focus on the ion-conducting properties of porous frameworks, which are especially advantageous for multivalent-ion systems. In addition, the design and synthesis strategies of MOF/COF materials are discussed and their applications as ion conductors are studied. Finally, future research directions to enhance MOF/COF performance as ion conductors and facilitate commercialization are outlined.

## 2 Ion Conducting Mechanisms of MOF/COF for Multivalent Ions

### 2.1 Unique Structures of MOF/COF for Multivalent Ions Conduction

MOF/COF materials possess ordered porous architectures with tunable pore sizes, enabling precise control over ion transport pathways. By selecting appropriate building blocks, pore dimensions can be tailored to provide uniform channels that accommodate multivalent ions with low activation energies.^50,51^ Additionally, incorporation of ionic functional groups (such as sulfonate or carboxylate groups) along the framework creates strong Coulombic interactions with multivalent ions, further reducing migration barriers.^52,53^ The robust covalent (or coordination) bonds in MOF/COF impart crystalline structures that remain stable during ion migration. Moreover, functionalized MOF/COF can selectively transport multivalent ions while excluding unwanted species, resulting in increased transference numbers.^54,55^ A high transference number minimizes concentration polarization, lowering internal resistance and enabling stable voltage under high-rate charging/discharging, as well as enhanced structural and electrochemical stability during long-term cycling. These unique structural features make MOF/COF excellent ion conductors with exhibiting low activation energies, high ionic conductivity, and sustained structural integrity for multivalent battery applications.

Compared to inorganic solids, rigid inorganic electrolytes exhibit limited flexibility, resulting in poor surface contact and high grain boundary resistance.^56,57^ For multivalent ions, the higher charge density induces stronger Coulombic interactions with inorganic lattices, which in turn reduce ion mobility. In contrast, the relatively flexible frameworks of MOF/COF can accommodate these Coulombic forces and facilitate ion migration (Figure 1). Some MOF/COF frameworks exhibit a “breathing” behavior with lattice expansion or contraction upon ion insertion which temporarily widens pore channels, lowering activation energy and enhancing ionic conductivity.^58^ MOF/COF materials offer continuous ion channels via multiple conduction pathways, including both interlayer and intralayer routes.

**Figure 1:**
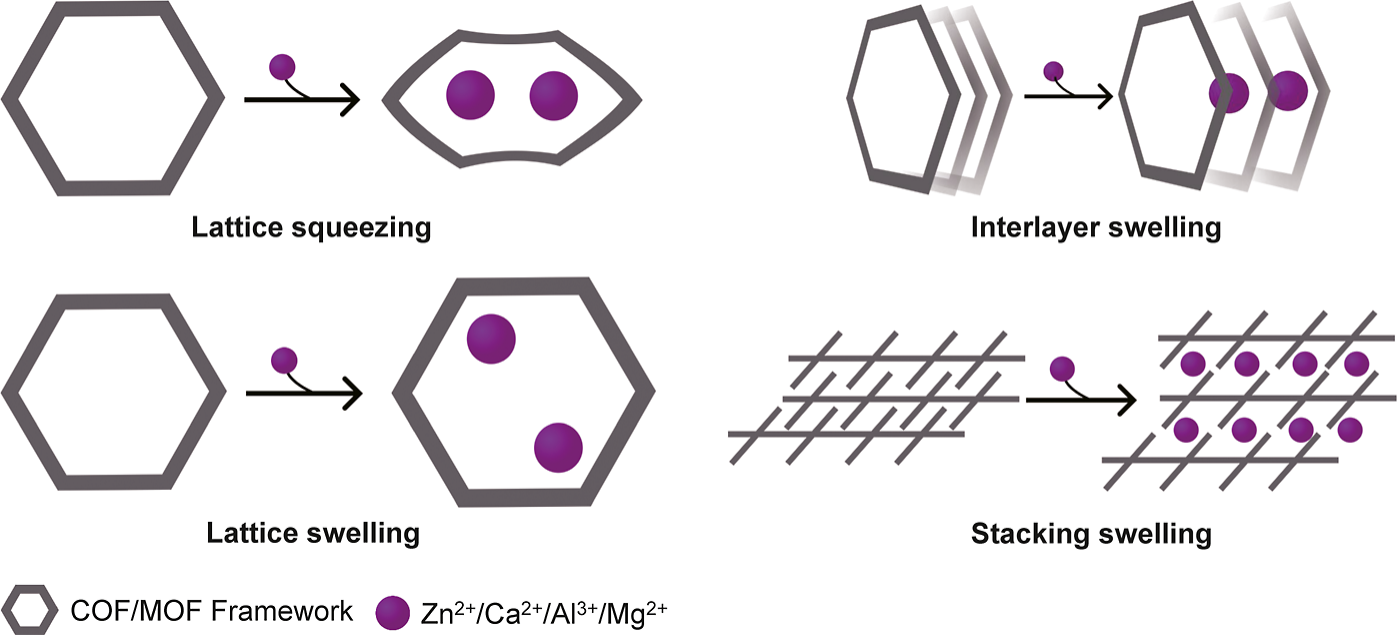
Scheme of unique structures of MOF/COF for multivalent ions conduction.

### 2.2 Ion Conducting Mechanisms of MOF/COF than Inorganic for Multivalent Ions

Several ion-conducting mechanisms can be observed in ion conductors. First, the hopping mechanism involves ions moving through the crystal lattice from one stable site to an adjacent one by overcoming an energy barrier.^59^ In this process, multivalent ions should have sufficient activation energy to overcome their local binding energy at the initial site. Second, vacancy-mediated diffusion occurs when ions exchange positions with lattice vacancies.^60^ An ion hops into a vacant site, leaving behind a new vacancy that can be occupied by a neighboring ion. Third, interstitial diffusion occurs as ions pass through small voids within the crystal lattice.^61^ Because multivalent ions are larger than monovalent ions, moving through narrow interstitial channels in a rigid lattice requires particularly high activation energies.

In oxide or sulfide inorganic conductors, interstitial sites are available for multivalent-ion migration. However, the void space is often too restricted to accommodate these larger ions. Moreover, multivalent ions encounter significant barriers at grain boundaries, which greatly increases grain-boundary resistance.^62^ As a result, conduction pathways become elongated and the activation energy for multivalent-ion transport can be high, hindering efficient ion conduction (Figure 2a).

**Figure 2:**
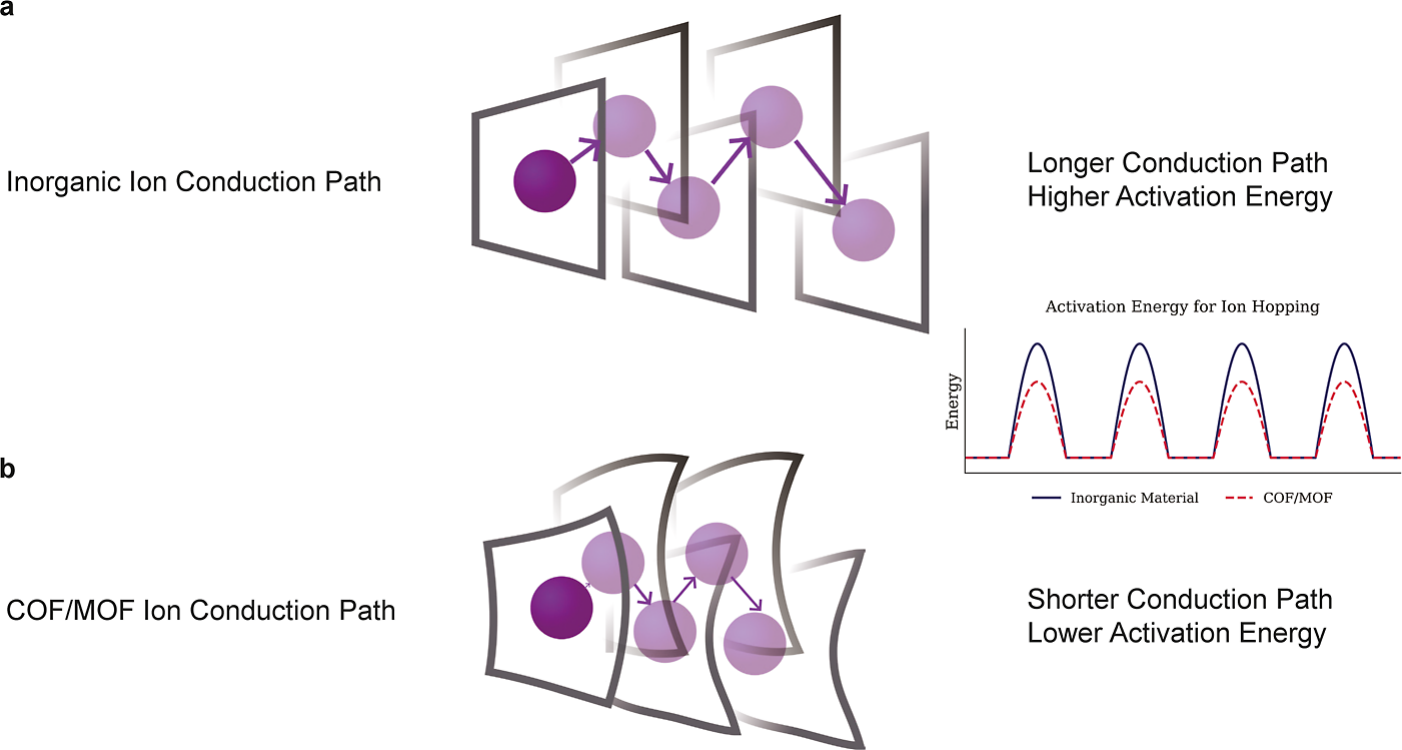
Multivalent ion conducting mechanisms of (a) inorganic and (b) MOF/COF materials.

In contrast, MOF/COF ion conductors benefit from several mechanisms that lower the energy barrier for multivalent-ion transport. First, their flexible organic–inorganic or organic–organic frameworks can deform slightly to accommodate ion hopping through the pore channels, reducing the activation energy.^63^ Second, the uniform nanopore architecture provides ample space for larger multivalent ions to migrate.^64^ The pore size and environment can be precisely tuned to enable selective ion transport and optimized ion mobility by offering an increased number of active sites. Third, multiple conduction pathways such as intralayer and interlayer directions make ions move through the lowest-energy route, further decreasing the overall migration barrier.^65^ When ionic functional groups are incorporated into the MOF/COF framework, strong Coulombic interactions with multivalent cations help stabilize the ion within the pore and shorten the effective migration distance, thereby lowering activation energy and facilitating faster ion transfer (Figure 2b). In 2D COFs, highly π-conjugated backbones delocalize electrons, creating a uniform electrostatic environment that further reduces ion-hopping barriers.^66^ This directional ion migration pathways can significantly enhance the ionic conductivity of multivalent ions.

## 3 Design and Synthesis of Multivalent Ion Conductive MOF/COF

### 3.1 Design of Multivalent Ion Conductive MOFs

Designing MOFs for efficient multivalent-ion conduction depends on several factors, including the shape of the ligand, the types of metal clusters, and the metal-to-ligand ratio required to construct the desired porous architecture (Figure 3). First, regarding ligand shape, planar π-conjugated linkers such as hexahydroxybenzene (HHB), 2,3,6,7,10,11-hexaiminotriphenylene (HITP), hexaaminobenzene (HAB), or 2,3,6,7,10,11-hexahydroxytriphenylene (HHTP), stack into uniform sheets and delocalize charge, thereby creating a homogeneous electrostatic environment with forming 2D structures that facilitates multivalent-ion migration.^67–73^ However, while a planar ligand is a necessary condition for forming 2D layered MOFs, it is not sufficient on its own. For example, UiO-66 (1,4-dicarboxybenzene (BDC) as ligand) and Mn­(BTC) MOF (1,3,5-benzenetricarboxylic acid (BTC) as ligand) are 3D structure.^74,75^ Second, metal clusters in MOFs play a critical role in determining their structure. Metal ions such as Cu^2+^, Co^2+^, and Ni^2+^ tend to form 2D layered MOFs by promoting in-plane connectivity between adjacent ligands. In contrast, metal clusters containing high-valent transition metals such as Ti^4+^, Cr^3+^, Al^3+^, and Fe^3+^ are typically found in 3D MOFs due to their ability to form multimetal clusters, such as Zr_6_O_4_(OH)_4_, which promote three-dimensional connectivity. Third, the ratio between metal clusters and organic ligands also plays a crucial role in determining the structure of MOFs. For example, by adjusting the ratio of Zn­(NO_3_)_2_ to BDC, both 2D layered and 3D cubic MOFs can be synthesized.^76^

**Figure 3:**
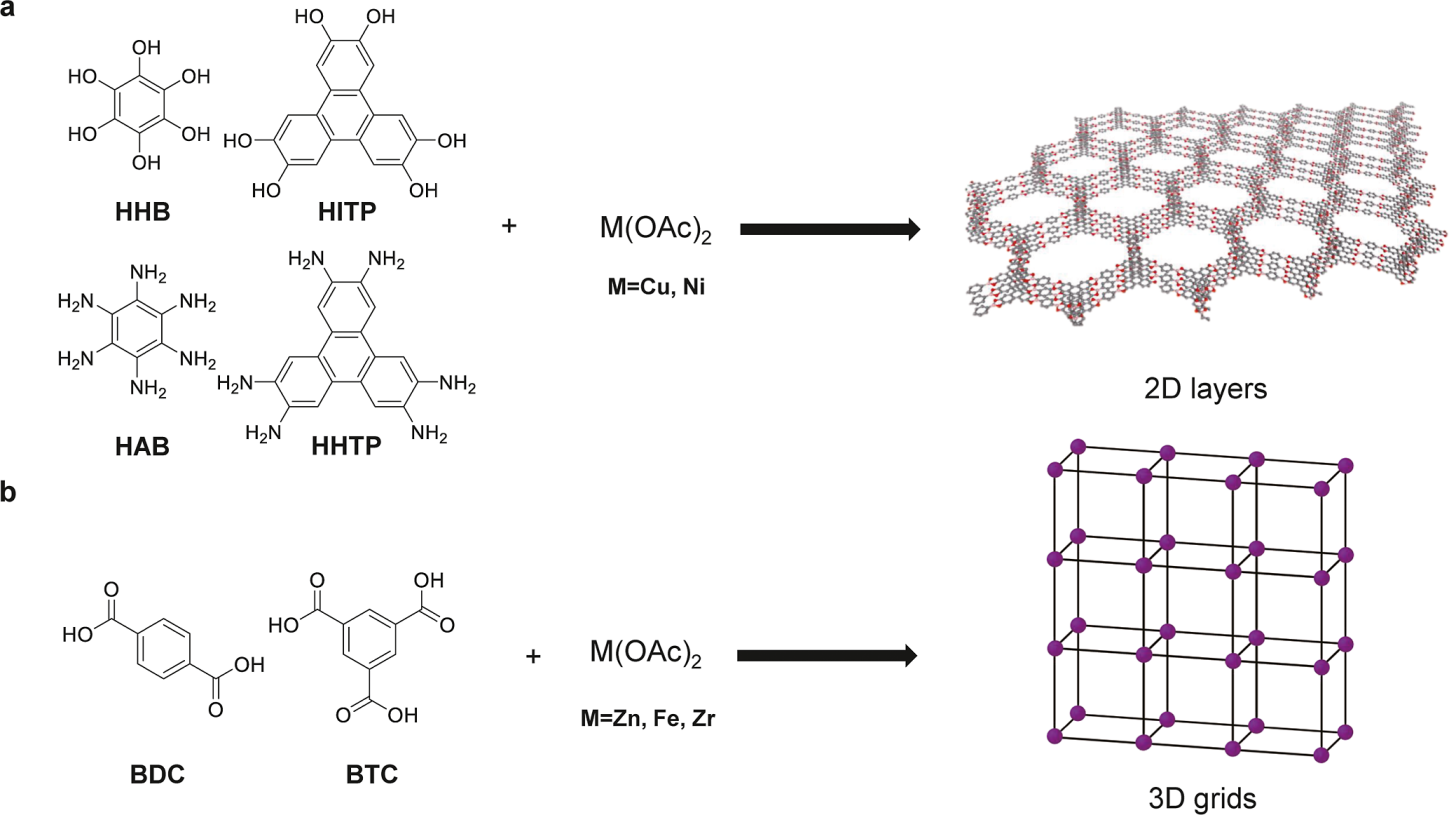
Design strategies of (a) 2D MOF and (b) 3D MOF as multivalent ion conductors.

Moreover, introducing ionic moieties (e.g., –SO_3_^–^ or –COO^–^) on organic ligand generates anionic sites within the layers, which serve as ion-hopping centers and lower the activation energy for ion transport.^77,78^ Furthermore, interlayer spacing can be tuned by varying ligand size or incorporating side chains.^79^ Careful selection of ligand length and geometry allows adjustment of cavity and channel dimensions, ensuring ample space for multivalent-ion diffusion.^80^ These design strategies balance structural stability with low activation energies, producing MOF frameworks optimized for high-performance multivalent-ion conduction.

### 3.2 Design of Multivalent Ion Conductive COFs

To design COFs for efficient multivalent-ion conduction, choosing organic monomers that form thermodynamically favorable covalent bonds to create a robust, porous framework is required.^81^ Pore dimensions can be tuned either by selecting monomers of appropriate size or by adjusting monomer ratios to accommodate bulky multivalent ions.^82^ Incorporating ionic groups into the framework provides additional ion-hopping sites and lowers the activation barrier for ion conduction.^53^

COF linkage chemistry can be tailored via monomer selection. Common linkage types include imine (Schiff-base), β-ketoenamine, and azine bonds.^83^ For example, imine linkages form when an amine-bearing monomer condenses with an aldehyde or ketone, generating reversible C=N bonds with error correction during crystallization. Depending on the chosen linkages and monomer geometries, COFs can be structured as either 2D layers or 3D networks, similar to MOFs.^84,85^

Embedding ionic functional groups is critical for enhancing multivalent-ion conductivity by shortening migration distances.^86^ This can be achieved either in two steps or in a single step. Two steps involve first synthesizing a nonfunctionalized COF and then grafting ionic moieties. On the other hand, a single step needs one monomer without functional groups and another monomer with functional groups (Figure 4). The one-step approach ensures uniform distribution of ionic sites, reduces synthesis time, and lowers costs by eliminating extra reagents and procedures, ultimately yielding COFs with reproducible, high multivalent-ion conductivity.

**Figure 4:**
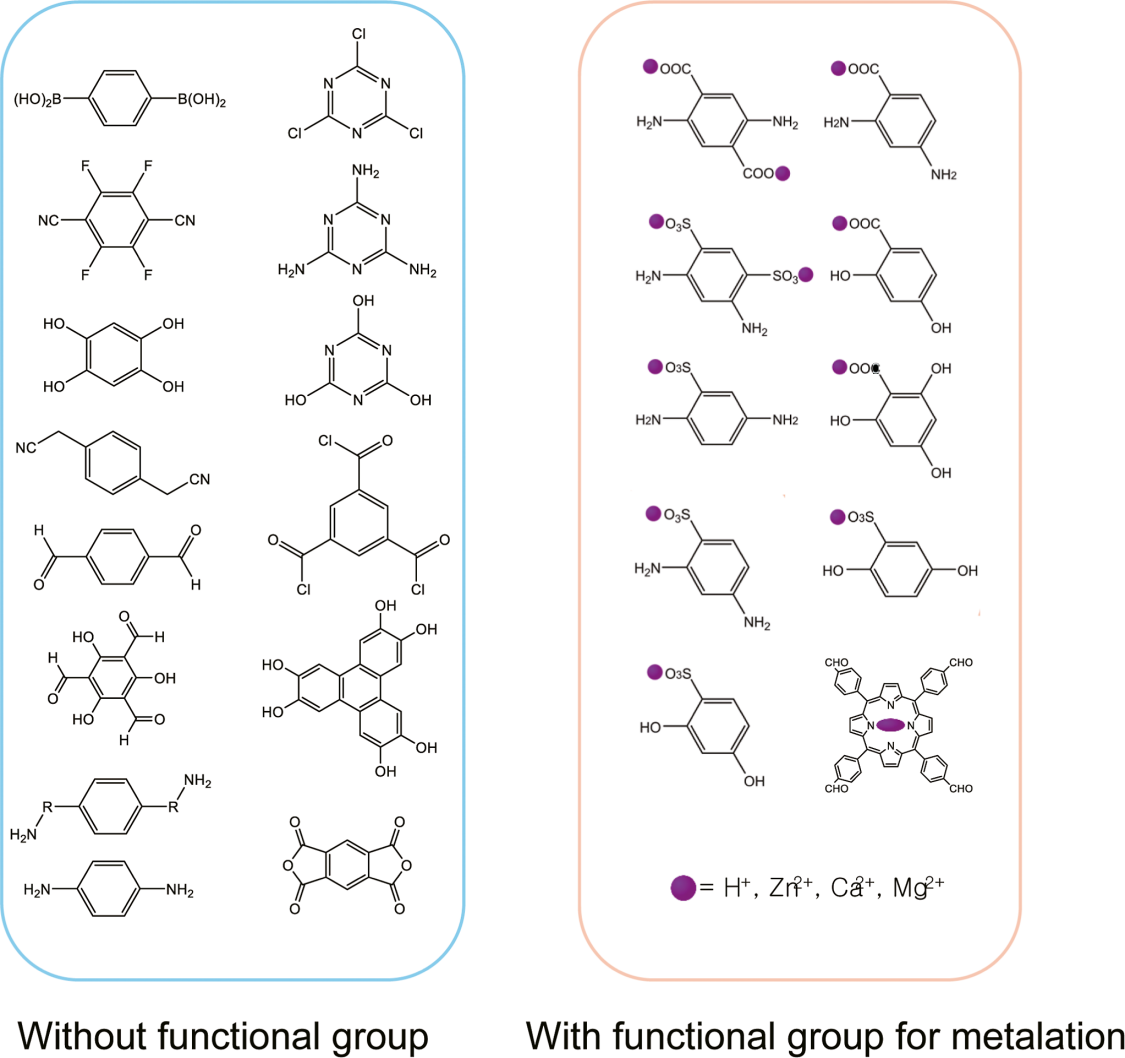
Building blocks without and with functional groups for covalent bond formation to design multivalent ion conductive COF.

### 3.3 Synthetic Methods of MOF/COF

MOF/COF materials can be synthesized using various methods (Figure 5). First, hydrothermal synthesis (Figure 5a), conducted in a sealed autoclave at temperatures above the solvent’s boiling point, accelerates dissolution and crystal growth via elevated pressure and temperature.^87^ Although hydrothermal conditions often yield highly crystalline materials, the requirement for high-pressure equipment involves complexity. Second, interfacial synthesis generates MOF/COF films or nanosheets at the boundary between two immiscible solvents (Figure 5b).^88,89^ One solvent dissolves the one monomer, and the other dissolves the other monomer. Framework nucleation and growth occur at the liquid–liquid interface. This method typically produces materials with excellent crystallinity and is well suited for membrane fabrication, but the limited interfacial area hinders scale-up for bulk production. By selecting the appropriate synthesis route based on available equipment, desired crystallinity, particle morphology, and environmental considerations, MOF/COF can be prepared effectively. Third, solution-based methods are widely used. In reflux synthesis, monomers are dissolved in a suitable solvent and heated under atmospheric pressure for several hours to days to promote framework assembly (Figure 5c).^90^ This straightforward setup allows easy monitoring of reaction progress, but achieving high crystallinity typically requires extended reaction times, and solvent costs can be substantial. Fourth, mechanical grinding is a rapid, solvent-free approach. In the mortar-and-pestle method, monomers are manually ground for several minutes (Figure 5d).^91^ This technique requires no specialized equipment, proceeds quickly, and eliminates solvent use, making it both eco-friendly and cost-effective. It is particularly useful for small-scale screening of candidate materials, although yields tend to be low. For more reproducible mechanical synthesis, ball milling can be employed: monomers and milling balls are loaded into a jar, and mechanical collisions induce bond formation, often with minimal or no solvent.^92^ Reaction times range from minutes to hours, and the method is suitable for large-scale batches. However, ball-milled products often require postsynthetic activation (e.g., heat treatment) to achieve high crystallinity.

**Figure 5:**
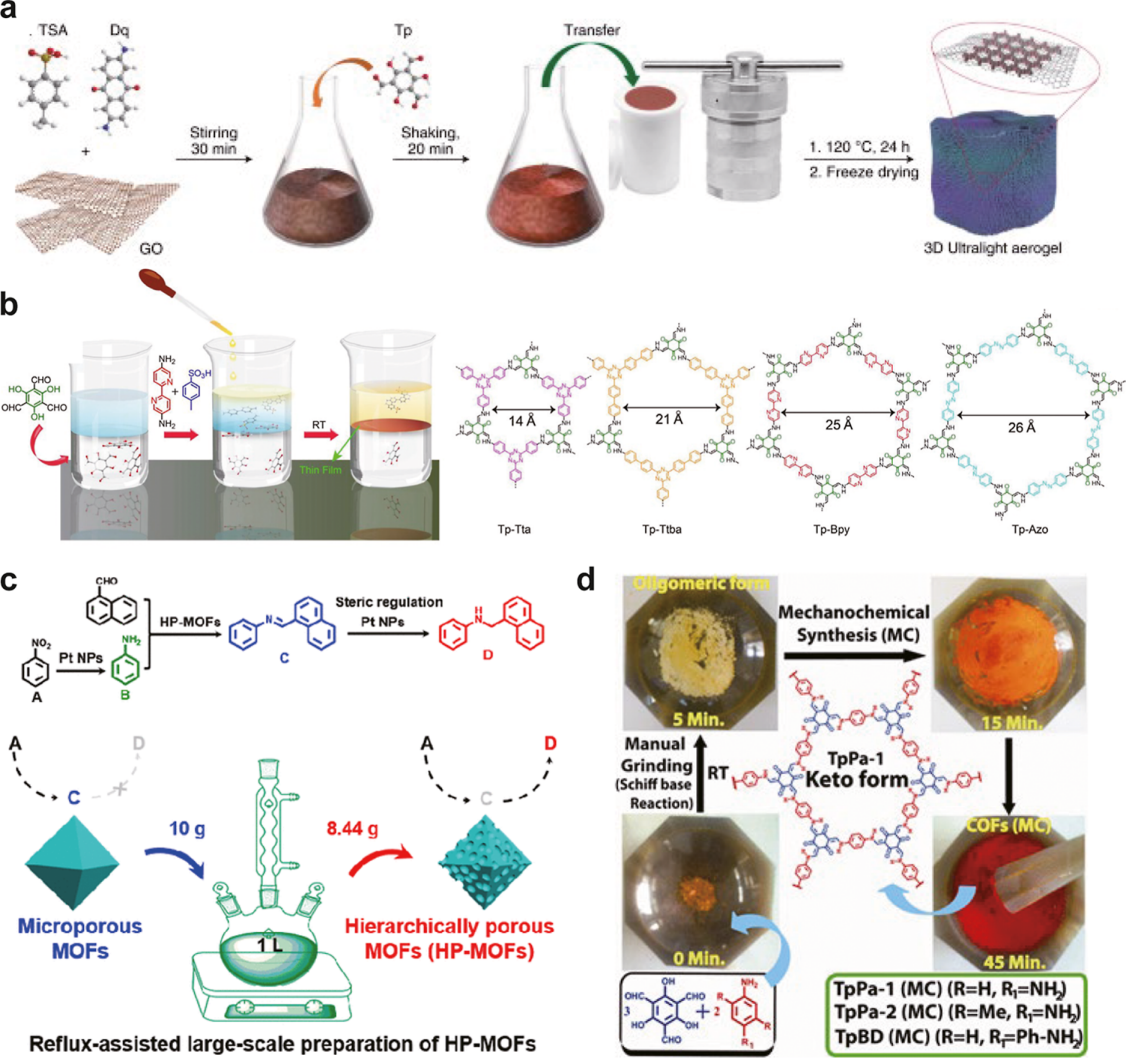
Various synthesis method for preparing MOF/COF. (a) Hydrothermal method (Reproduced with permission from ref 87. Copyright 2020 Springer Nature), (b) interfacial synthesis method (Reproduced from ref 89. Copyright 2017 American Chemical Society), (c) reflux method (Reproduced from ref 90. Copyright 2021 American Chemical Society), and (d) mechanical grinding method (Reproduced from ref 91. Copyright 2013 American Chemical Society).

## 4 Applications of MOF/COF as Multivalent Ion Conductors

MOF/COF frameworks can serve as multivalent-ion conductors in several key applications. First, they can be utilized as solid electrolytes for multivalent batteries. Continuous nanopore channels decorated with anchored ionic groups enable MOF/COF frameworks to accommodate bulky cations (e.g., Zn^2+^, Mg^2+^, Ca^2+^, Al^3+^) and provide uninterrupted ion-transport pathways. The fixed anionic sites offer additional hopping sites, thereby lowering the activation energy for ion migration. As a result, MOF/COF solid electrolytes can achieve room-temperature conductivities of 10^–6^–10^–4^ S cm^–1^. The analysis techniques about ion-conducting properties such as ion conductivity, activation energy, or transference number can be studied in our previous review literature.^86^ Chen et al. synthesized a tetraphenylborate-based anionic MOF loaded with MgI_2_, CaI_2_, or ZnI_2_ and they exhibited high conductivities of 0.328 mS cm^–1^, 0.13 mS cm^–1^, and 0.61 mS cm^–1^, respectively, with relatively low activation energies (Figure 6).^41^ Sadakiyo et al. developed a COF containing Mg^2+^ carriers and poly­(ethylene oxide) chains in its channels and it demonstrated a superionic conductivity of 1.8 × 10^–4^ S cm^–1^ (Figure 7).^39^ Blending MOF/COF particles with polymer matrices (e.g., PEO or PVDF-HFP) further enhances mechanical strength, allowing these composites to withstand stack pressure or bending in solid-state cells. Second, MOF/COF can serve as membranes for multivalent flow batteries. When employed as ion-selective membranes, MOF/COF membranes can conduct multivalent cations while blocking active species. This selectivity reduces concentration polarization and species crossover, improving Coulombic efficiency and extending cycle life even under high-current operation. Third, MOF/COF can be used as electrode coating materials for interface regulation.^93^ Thin MOF/COF coatings on multivalent metal or intercalation electrodes can regulate ion flux and stabilize the solid–electrolyte interphase. The porous frameworks mediate ion insertion/extraction kinetics and prevent localized overplating, enabling prolonged cycling with reduced capacity fade. In summary, the combination of tunable pore architectures, anchored ionic functionalities, and mechanical adaptability allows MOF/COF materials to address critical challenges in multivalent energy storage devices.

**Figure 6:**
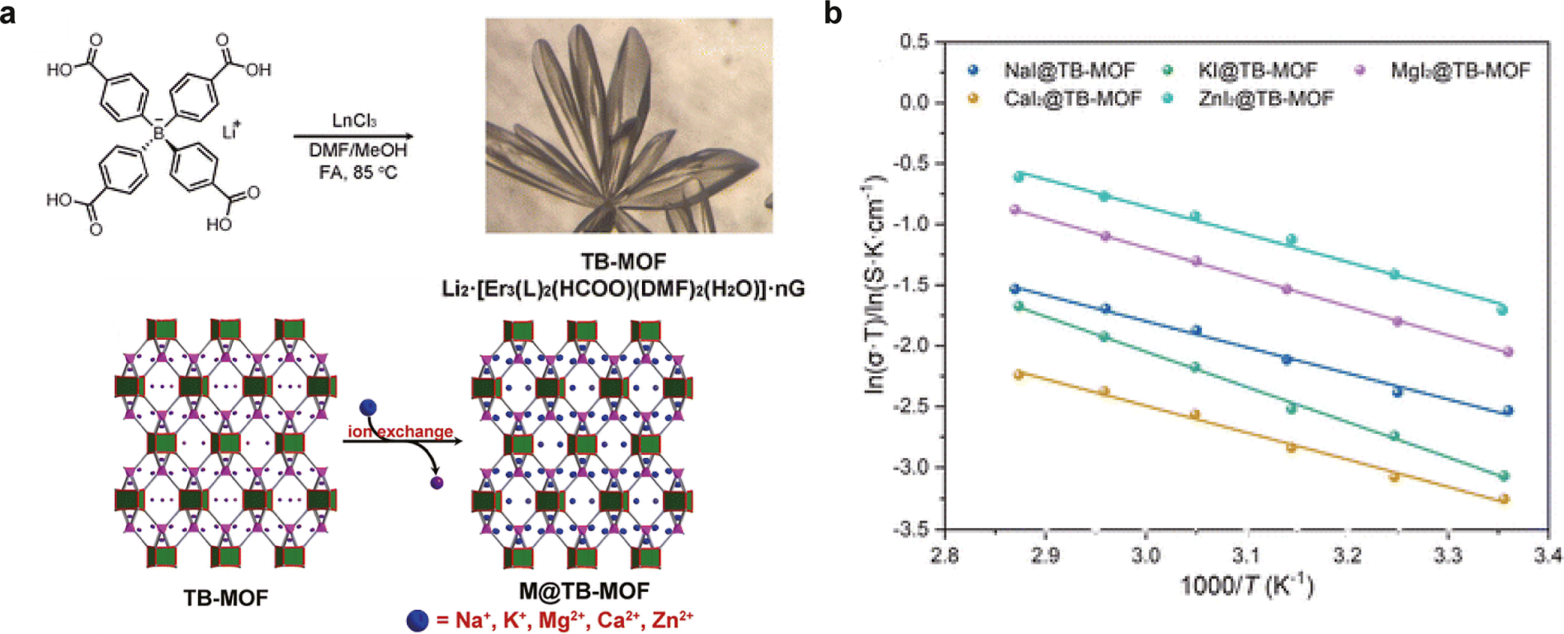
Example of multivalent ion conducting MOF. (a) Schematic illustration of the grafting of other metal iodides into TB-MOF. (b) Arrhenius plots for the ionic conductivity of NaI@TB-MOF, KI@TB-MOF, MgI_2_@TB-MOF, CaI_2_@TB-MOF, and ZnI_2_@TB-MOF. (Reproduced from ref 41. Available under a CC-BY 3.0 license. Copyright 2024 Xuenian Chen et al.).

**Figure 7:**
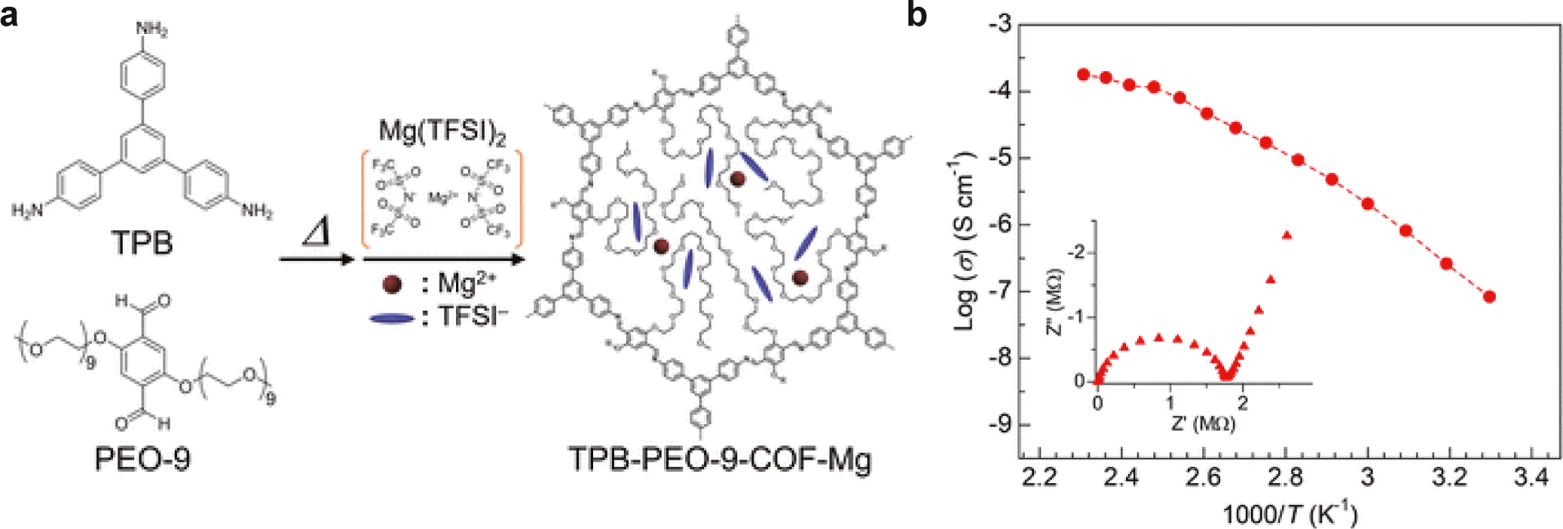
Example of multivalent ion conducting COF. (a) Schematic illustration of the synthesis of TPB-PEO-9-COF-Mg. (b) Temperature dependence (30–160 °C) of ionic conductivity of TPB-PEO-9-COF-Mg under N_2_. The inset shows an example of Nyquist plots (50 °C). (Reproduced from ref 39. Available under a CC-BY-NC 3.0 license. Copyright 2024 Masaaki Sadakiyo et al.).

## 5 Conclusion

In this review, the fundamental ion-conducting mechanisms, structural design principles, synthetic methodologies, and applications of MOF/COF frameworks as multivalent-ion conductors were discussed. By combining ordered nanopore architectures with anchored ionic sites, MOF/COF materials can achieve reduced activation energies and elevated ionic conductivities. We outlined design strategies for tuning pore dimensions, enhancing framework flexibility, and introducing functional groups to optimize multivalent-ion transport and reviewed several synthesis approaches. Additionally, the potential roles of MOF/COF materials as solid electrolytes and membranes in multivalent-ion batteries were discussed.

Despite reports of promising ionic conductivities in MOF/COF materials, no study to date has demonstrated a functioning multivalent-ion battery cell employing a MOF/COF ion conductor. This gap arises from several challenges such as interfacial instability with multivalent metal electrodes, insufficient mechanical integrity to withstand the high stack pressures during cell assembly, and incomplete ionic percolation in scaled-up membranes. Additionally, many MOFs and COFs suffer from limited chemical and electrochemical stability under harsh battery operating conditions, such as low-pH or high-voltage environments. The narrow electrochemical stability window of some frameworks, potential redox activity of metal nodes or organic linkers, and vulnerability to hydrolysis further complicate their practical implementation. Moreover, the lack of scalable, robust membrane fabrication techniques such as the integration of MOF/COF particles into continuous, defect-free, and flexible films remains a major barrier to full-cell demonstration.

Future research should address these obstacles by pursuing interfacial engineering strategies, developing mechanically reinforced composites, establishing scalable fabrication methods, and performing integrated cell-level testing. Long-term stability assessments and further enhancements in ionic conductivity will also be crucial. Addressing these areas will be essential to realize MOF/COF materials as practical ion conductors in next-generation multivalent-ion energy storage devices.
